# The associations of risk of cardiovascular disease with development stages of diabetes in Chinese population: findings from a retrospective cohort study in QuZhou city

**DOI:** 10.1186/s12902-024-01544-1

**Published:** 2024-02-02

**Authors:** Qi Wang, Zhijuan Gan, Qing Gao, Meng Wang, Bingdong Zhan

**Affiliations:** 1 Zhejiang Chinese Medicine University, 548 Binwen Road, Binjiang District, 310053 Hangzhou, Zhejiang Province China; 2 Quzhou Center for Disease Control and Prevention , 154 Xi’an Road, Kecheng District, 324003 Quzhou , Zhejiang Province China; 3 Zhejiang Provincial Center for Disease Control and Prevention, 3399 Binsheng Road, Hangzhou, China

**Keywords:** Fasting plasma glucose, Prediabetes, Diabetes, Cardiovascular disease

## Abstract

**Background:**

Risk analysis is an important area of research in diabetes and cardiovascular disease (CVD), both of which have significant global health burdens. Although there is evidence that patients with prediabetes and diabetes mellitus may have an increased risk of CVD, few studies have been conducted in mainland China.

**Methods:**

This retrospective cohort study utilized data from the Quzhou City Resident Health Information System and the Zhejiang Province Chronic Disease Surveillance System in China. Prediabetes and diabetes mellitus were the exposure interests, and the outcome event was defined as the onset of cardiovascular and cerebrovascular disease (including coronary heart disease and stroke). The start date of the study was January 1, 2015, and the follow-up deadline was December 31, 2020. Multivariate Cox proportional hazard model were used to assess the associations among prediabetes, diabetes, and CVD risk. Hazard ratios (HRs) and 95% confidence intervals (CIs) were calculated. Our study used follow-up time as the time scale, while adjusting for age, sex, physical activity, smoking, alcohol consumption, BMI in the models Sensitivity analyses were conducted to assess the stability of the results, by excluding participants who smoked and drank alcohol, participants who developed CVD in the first year of follow-up.

**Results:**

In total, 138,970 participants were included in our study, with a mean follow-up of 5.8 years. The mean age of the participants was 58.82 ± 14.44 years, with 42.79% (*n* = 59,466) males and 57.21% (*n* = 79,504) females. During the study period 4357 cases of CVD were recorded. Participants with prediabetes (*P* = 0.003) and diabetes (*P* < 0.001) had a higher risk of CVD than those who were Normal (HR [95% CI]: 1.14 [1.05–1.24]; 1.68 [1.55–1.81], respectively). Prediabetes and patients living with diabetes had a 14% and 68% increased risk of CVD, respectively. The results of the sensitivity analyses were consistent with those of the main analyses after excluding those who developed CVD within one year of follow-up and those who were concurrent smokers or alcohol drinkers.

**Conclusions:**

Our research found that prediabetes is significantly associated with the risk of diabetes and CVD.

**Supplementary Information:**

The online version contains supplementary material available at 10.1186/s12902-024-01544-1.

## Introduction

Cardiovascular diseases (CVD) are a group of heart and blood vessel disorders that include coronary heart disease, cerebrovascular disease, rheumatic heart disease, congenital heart disease and deep vein thrombosis and pulmonary embolism. CVD is the leading cause of premature mortality and morbidity worldwide. According to WORLD HEART REPORT (2023), the number of deaths due to CVD (e.g., myocardial infarction or stroke) increased from 12.1 million to 18.6 million globally between 1990 and 2019 [1]. Simultaneously, CVD places a heavy burden on families and the economy [2]. Therefore, CVD prevention is a public health issue in China and worldwide.

Diabetes mellitus (DM) is a chronic metabolic disease caused by impaired insulin secretion or function and is characterized by chronic hyperglycemia [3]. Long-term abnormalities in glucose metabolism can lead to vascular lesions and subsequently induce cardiovascular disease [4]. Studies have proposed that type 2 diabetes mellitus (T2DM) is a coronary heart disease equivalent (risk equivalent), that is, patients with T2DM have a similar risk of future myocardial infarction and CVD deaths to those without T2DM who have had a previous heart attack [5]. Prediabetes is an important stage in the transition from a healthy state with normal blood glucose to diabetes [6] and is characterized by impaired fasting glucose (IFG) and impaired glucose tolerance (IGT). Studies in Korea [7], Australia [8], and Europe [9] found that CVD risk is already present in prediabetes. CVD and diabetes comorbidities are significant mortality risks; CVD is the most common cause of death in patients with T2DM [10]. Worldwide, approximately 32.2% of patients with T2DM are affected by CVD [11]. Studies have shown that adults with T2DM have a 2- to 4-fold higher risk of CVD and death than those without T2DM [12]. In addition to the inevitable increase in deaths in people with diabetes, the combined mortality rate of diabetes and CVD has almost doubled [12].

T2DM is associated with clustering risk factors for CVD. Research shows that the prevalence of hypertension in adult patients with diabetes is 77 -87%, the prevalence of elevated low-density lipoprotein cholesterol (LDL-C) is 74 -81%, and the prevalence of obesity is 62 -67% [13]. T2DM is a major controllable risk factor for CVD; however, it is not the only risk factor. The vascular complications of DM are associated with various risk factors, including dyslipidemia, hypertension, obesity, smoking, age, other metabolic diseases, and systemic inflammation [14]. Although the effects of these risk factors on the cardiovascular system are known, the extent to which hyperglycemia affects CVD requires further in-depth studies in China. This study aimed to analyze the link among prediabetes, diabetes, and cardiovascular risk, which is essential to reduce the risk of CVD.

## Methods

### Participants and setting

This retrospective cohort study was designed in accordance with the Specifications for Reporting of Observational Studies (STROBE). Since 2015, information entry into the Quzhou City Resident Health Information System has been gradually improving, and the occurrence of CVD after 2020 may have been affected by the COVID-19 Pneumonia. Therefore, we chose to collect information from health checkups for residents in Quzhou City, Zhejiang Province, China, from January 1, 2015, to December 31, 2020. Our study followed participants based on the Quzhou City Resident Health Information System, which records resident health, hospital treatment, and death registration information, and we were able to comprehensively assess each resident’s real-time health events. Information on cardiovascular disease for each participant was obtained from the Zhejiang Provincial Chronic Disease Surveillance System. At baseline, each participant was medically examined by a physician at a local rural hospital and provided data on characteristics, including gender, age, height, weight, blood pressure, marital status, education, and lifestyle (e.g., smoking, alcohol consumption, and physical activity), which were entered into the Resident Health Record Information Database. The participants were closed to follow-up if they developed cardiovascular diseases (coronary heart disease or stroke). Inclusion criteria: Participants who voluntarily participated in the medical examination of the population were included. Participants with (1) duplicate identity card numbers, (2) missing Fasting plasma glucose measurements, (3) fasting glucose less than 3.9 mmol/L, and (4) a follow-up time of less than or equal to 0 (i.e., a history of diagnosed cardiovascular disease at baseline: information from the Chronic Disease Surveillance System) were excluded from analysis.

### Exposure definitions

#### Fasting plasma glucose (FPG) assessment

The participants fasted overnight and blood samples were collected between 07:30 and 09:00. Blood samples were collected and analyzed within 4 h. FPG (Beckman Coulter AU5800) was analyzed using a biochemical autoanalyzer.

#### Definition of diabetes, prediabetes

Diabetes is a chronic disease that occurs when the pancreas does not produce enough insulin or when the body is unable to efficiently use produced insulin. Prediabetes is the transitional stage before the onset of diabetes and includes IFG, IGT, and a combination of the two (IFG + IGT), an intermediate hyperglycemic state between normoglycemia and diabetes. Our study classified FPG levels into three categories based on the criteria published by the American Diabetes Association in 2018 [15]: diabetes (FPG ≥ 7.0 mmol/l or self-reported diabetes); prediabetes (IFG [5.6 mmol/l ≤ FPG < 7.0 mmol/l]) and normal FPG (3.9 mmol/l ≤ FPG < 5.6 mmol/l) [16].

### Outcome definition

CVD: a collective term for cardiovascular and cerebrovascular diseases referring to ischemic or hemorrhagic disorders occurring in the heart, brain, and systemic tissues due to hyperlipidemia, blood viscosity, atherosclerosis, and hypertension. The occurrence of CVD in each participant was determined using the Zhejiang Province Chronic Disease Surveillance System.

### Covariate definitions

Body mass index [17] was calculated as weight (kg)/height^2^ (m^2^). Smoking was defined as having smoked continuously or cumulatively for more than 6 months, smoking at least 1 cigarette per day, and currently still smoking; otherwise, it was defined as never having smoked. Former smokers who had quit smoking and had done so for more than 6 months were defined as having quit smoking. Occasional drinking was defined as less than or equal to one drink per week, and regular drinking was defined as drinking more than one drink per week (excluding one drink), but less than daily drinking. Smoking and drinking at a very low frequency due to socialization were judged as non-smoking or non-drinking, as appropriate. Physical activity was estimated by weekly exercise time based on exercise type and duration.

### Statistical analysis

Data were statistically analyzed using R4.3.2 (R Foundation for Statistical Computing, Vienna, Austria) and Microsoft Excel (Microsoft Corporation, Redmond, WA, USA). In the descriptive statistics section of this study, data for normally distributed continuous variables were expressed as mean ± standard deviation, and categorical variables were described using frequencies (percentages). Multivariate Cox proportional risk models were used to estimate hazard ratios (HRs) and 95% confidence intervals (CIs) for the association between prediabetes, diabetes, and CVD risk.

This study used the follow-up time as the time scale, while adjusting for age, gender, physical activity, smoking, alcohol consumption, and BMI in the model. CVD within the first year of follow-up may not be related to exposure factors, other than the fact that smokers and alcohol drinkers have a higher risk of CVD. Sensitivity analyses excluded the effects of smoking, alcohol consumption, having less than 1 year of follow-up, or having CVD in the first year on CVD risk analyses and assessed whether the results were robust (*P* < 0.05, considered statistically significant).

To adjust for potential confounders (age, gender, physical exercise, smoking, alcohol consumption, and BMI), we constructed four continuity models. In Model 1, no variables were adjusted. Model 2 was adjusted for age and gender. In Model 3, age, gender, physical exercise, smoking, and alcohol consumption were adjusted. In Model 3, BMI was added for adjustment.

The Cox proportional risk model was implemented by the survival package of the R software, with a test level of a = 0.05 and all statistical tests were based on a two-sided 5% significance level. HR was used to estimate the multiplicative change in the risk of an outcome event due to the presence of an exposure factor. When HR > 1, it indicates that there is a positive correlation between the exposure factor and the disease; when HR < 1, it indicates that there is a negative association; when HR = 1, it indicates no association between exposure factor and disease.

## Results

### Participant characteristics

**Fig. 1:**
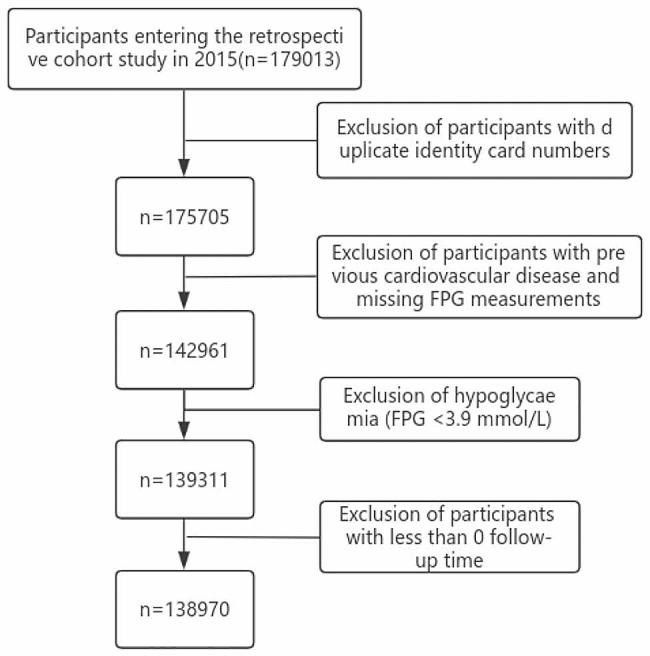
Flow chart of retrospective cohort study

Of 179,013 participants enrolled in the retrospective cohort, the final sample size for inclusion in the study was 138,970. A flowchart of the study is shown in Fig. 1. The mean age of participants without CVD at baseline was 58.82 ± 14.44 years. A total of 105,310 patients had normal fasting glucose levels, 18,615 had prediabetes (IFG), and 15,045 had diabetes. There were 59,466 (42.79%) males and 79,504 (57.21%) females. Among the participants, 77.56% never smoked, 78.33% never drank alcohol, and about 7/10 never exercised. The average BMI was of the participants 23.25, which was within the normal range based on the standard in China (18.5–23.9 kg/m^2^) (Table 1).

**Table 1:** Baseline characteristics of the 2015 medical examination population in Quzhou, China

	N	Normal	Prediabetes	Diabetes
Number	138,970	105,310	18,615	15,045
Incidence density (person-years)		0.00480	0.00603	0.00913
**Age, mean (SD)**	58.82 ± 14.44	57.66 ± 15.13	62.01 ± 11.51	62.98 ± 10.99
**Genders**				
Male(%)	59,466 (42.79)	46,299 (43.96)	7321 (39.33)	5846 (38.86)
Female(%)	79,504 (57.21)	59,011 (56.04)	11,294 (60.67)	9199 (61.14)
**BMI(mean (SD))**	23.25 ± 20.74	23.11 ± 19.58	23.61 ± 26.80	23.81 ± 20.08
**Physical exercise**				
No(%)	103,274 (74.31)	76,366 (72.52)	14,660 (78.75)	12,248 (81.41)
Infrequent(%)	15,342 (11.04)	12,069 (11.46)	1983 (10.65)	1290 ( 8.57)
Once a week(%)	7202 ( 5.18)	6138 ( 5.83)	700 ( 3.76)	364 ( 2.42)
Everyday(%)	12,471 ( 8.97)	10,178 ( 9.66)	1209 ( 6.49)	1084 ( 7.21)
**Smoking status**				
Never(%)	107,790 (77.56)	81,584 (77.47)	14,559 (78.21)	11,647 (77.41)
Quit(%)	5890 ( 4.24)	4181 ( 3.97)	905 ( 4.86)	804 ( 5.34)
Smoking(%)	24,362 (17.53)	18,725 (17.78)	3089 (16.59)	2548 (16.94)
**Drinking status**				
Never(%)	108,857 (78.33)	82,556 (78.39)	14,584 (78.35)	11,717 (77.88)
Occasional(%)	9490 ( 6.83)	7234 ( 6.87)	1244 ( 6.68)	1012 ( 6.73)
Regular(%)	6072 ( 4.37)	4753 ( 4.51)	763 ( 4.10)	556 ( 3.70)
Everyday(%)	12,943 ( 9.31)	9481 ( 9.00)	1850 ( 9.94)	1612 (10.71)

### Association between prediabetes and cardiovascular disease risk

**Fig. 2:**
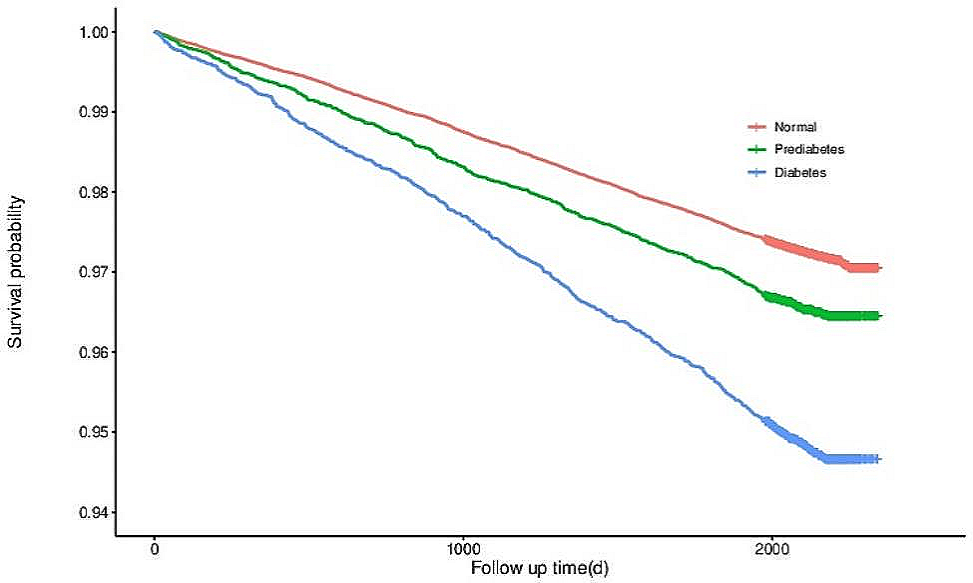
Survival analysis of participants with normal FPG, prediabetes, diabetes mellitus

During the study period, 4,357 participants (2,923, 647, and 787 with normoglycemia, prediabetes, and diabetes) developed CVD during a mean follow-up period of 5.8 years. We chose participants with normal fasting glucose as the reference category, and after adjusting for potential confounders (including age, gender, smoking, alcohol consumption, physical activity, and BMI factors), the risk of developing cardiovascular and cerebral vascular disease was statistically significant in the populations with prediabetes (HR 1.14; 95% CI 1.05, 1.24) and diabetes (HR 1.68; 95% CI 1.55,1.81) compared with the participants with normal fasting glucose (*P* < 0.05). The risk of CVD was significantly increased by 14% (95% CI: 5-24%) in participants with prediabetes and 68% (95% CI: 55-81%) in participants with diabetes (Table 2). Survival plots are shown in Fig. 2.

**Table 2:** Adjusted Hazard Ratio (95% CI) for cardiovascular disease in prediabetes, diabetes

	N(%)	CVD(%)	Model 1	Model 2	Model 3	Model 4
Normal	105,310(75.78)	102,387(76.06)	1.00[Ref]	1.00[Ref]	1.00[Ref]	1.00[Ref]
Prediabetes	18,615(13.39)	17,968(13.35)	1.26 (1.15,1.37)*	1.11 (1.02,1.21)*	1.14 (1.05,1.24)*	1.14 (1.05,1.24)*
Diabetes	15,045(10.83)	14,258(10.59)	1.90 (1.76,2.06)*	1.63 (1.50,1.76)*	1.68 (1.55,1.82)*	1.68 (1.55,1.81)*

FPG normal was selected as the reference category. Model 1 does not adjust for covariates, Model 2 adjusts for age and gender, Model 3 adjusts for age, gender, physical activity, smoking, alcohol consumption, Model 4 adjusts for age, gender, physical activity, smoking, alcohol consumption, BMI

*Significant results

### Interactions

The interaction showed statistically significant differences for gender, BMI, physical exercise, smoking (*P* < 0.05), age (*P* < 0.001), and alcohol consumption (*P* = 0.037). This indicates that there was no interaction between prediabetes and diabetes based on gender, BMI, physical exercise, and smoking, but there is an interaction between prediabetes and diabetes based on age and alcohol consumption. Table 3.

**Table 3:** Diabetes staging based on the interaction of age, gender, BMI, Physical exercise, smoking and drinking

	Chisq	*P*
Interaction of diabetes staging and age likelihood ratio	31.90	< 0.001
Interaction of diabetes staging and gender Likelihood Ratio	2.78	0.249
Interaction of diabetes staging and BMI Likelihood Ratio	0.25	0.882
Interaction of diabetes staging and Physical exercise Likelihood Ratio	11.42	0.179
Interaction of diabetes staging and Smoking Likelihood Ratio	5.40	0.494
Interaction of diabetes staging and Drinking Likelihood Ratio	16.41	0.037

### Sensitivity analysis

In order to assess the impact of CVD occurrence in the first year, as well as the impact of smoking, and alcohol consumption factors, which are strongly associated with CVD, participants with CVD within one year of follow-up or those who also smoked and drank alcohol were excluded. Diabetes was found to be present, albeit at a slightly different HR, after adjusting for potential confounders, including age, gender, smoking, alcohol consumption, physical activity, and BMI. The association between prediabetes and risk of cardiovascular disease was broadly consistent (Table 4).

**Table 4:** Sensitivity analysis: CVD Hazard Ratio adjusted for risk factors (95% CI)

	Model 1	Model 2	Model 3	Model 4
Exclusion of people with CVD in the first year of follow-up				
Normal	1.00[Ref]	1.00[Ref]	1.00[Ref]	1.00[Ref]
Prediabetes	1.22(1.11,1.34)*	1.09(0.99,1.19)	1.11(1.01,1.22)*	1.11(1.01,1.22)*
Diabetes	1.91(1.75,2.08)*	1.64(1.50,1.78)*	1.69(1.55,1.84)*	1.69(1.55,1.84)*
Excluding both smokers and drinkers				
Normal	1.00[Ref]	1.00[Ref]	1.00[Ref]	1.00[Ref]
Prediabetes	1.32(1.19,1.47)*	1.16(1.04,1.29)*	1.19(1.07,1.32)*	1.19(1.07,1.32)*
Diabetes	2.05(1.86,2.26)*	1.73(1.57,1.91)*	1.79(1.62,1.97)*	1.79(1.62,1.97)*

Model 1 does not adjust for covariates, Model 2 adjusts for age and gender, Model 3 adjusts for age, gender, physical activity, Model 4 adjusts for age, gender, physical activity, BMI

*Significant results

## Discussion

In this retrospective cohort study including 138,970 participants, we examined the associations among prediabetes, diabetes, and CVD risk in a Chinese population and found that diabetes and prediabetes increased the risk of CVD. The HRs were 1.14 (95% CI 1.05,1.24) and 1.68 (95% CI 1.55,1.81), respectively, after adjusting for potential confounders. The risk of prediabetes and diabetes significantly increased CVD risk by 14% (95% CI 5%, 24%) and 68% (95% CI 55%, 81%), respectively. Given the annual increase in the number of people with diabetes and prediabetes in China, even a slight increase in CVD risk has important public health implications at the population level.

### Comparison with other studies

Our findings on the relationship among prediabetes, diabetes, and CVD risk at baseline are largely consistent with those of other studies on this topic [10, 18, 19]. However, there are still conflicting results between different studies regarding whether the risk of developing diabetes can be equated with the risk of CVD, and whether there is a strong association between diabetes and CVD. Prospective studies from Denmark and Finland have shown that patients without diabetes with previous myocardial infarction have a similar risk of CVD death compared to patients with diabetes without myocardial infarction [20–21]. Stamler presented a different view; although the risk of CVD death in patients with diabetes is three times higher than that in patients without diabetes, diabetes is not a cardiovascular risk equivalent, but a major risk factor for the development of CVD [22]. Moreover, it has been found that the risk of CVD was even 43% lower in patients without diabetes compared to those with undiagnosed diabetes and previous myocardial infarction [23]. Overall, our analysis confirmed that DM increased the risk of CVD by 68% compared to patients with normal FPG.

Our findings also showed that even in the prediabetes stage, there was a significant 14% increase in the risk of CVD compared to those with normal fasting glucose levels, and this association remained statistically significant after multivariate adjustment. This finding is consistent with the results of previous studies conducted in Korea [24], the United States [25], and Singapore [26]. There was a quantitative relationship between CVD and degree of hyperglycemia [27–28]. Because the IFG measures only one blood glucose value, the results of some studies have been inconsistent. In the Jackson Heart Study, prediabetes was not associated with an increased risk of CVD [29]. An occupational cohort study in Japan showed that the association between prediabetes and CVD was not significant when prediabetes was defined solely based on the IFG [30]. The reason for these inconsistent results is unclear and may be due to differences in subject characteristics, study design, and sample size.

CVD is a chronic disease that requires a relatively long period between the development of risk factors and its onset. It is possible that IFG is not associated with an increased risk of CVD over a relatively short period [31]. Prediabetes, defined by the IFG, is an inexpensive and convenient monitoring method to limit the progression to diabetes and reduce vascular complications associated with hyperglycemia [32]. Further prospective studies are needed to investigate the effect of prediabetes on the risk of CVD. In the context of the increasing risk of CVD in China and worldwide, the present study showed that diabetes is an independent CVD risk factor, and that persistent prediabetes also increases the incidence of CVD.

### Interaction and sensitivity analyses

Our study performed an interaction analysis that revealed an interaction between age, alcohol consumption, and diabetes. To test the robustness of the results and potential variability, we performed sensitivity analyses excluding CVD within the first year of follow-up to reduce potential reverse causality. The findings were consistent with the total population - alcohol consumption and smoking are the two main risk factors for CVD [33].

### Mechanisms of CVD risk already present in early diabetes

The mechanisms underlying the development of early CVD in patients with diabetes are complex and remain unclear. It is currently believed that this may be related to various factors, such as insulin resistance, hyperglycemia, and vascular endothelial dysfunction. Studies have shown that in the prediabetes stage, the risk of CVD increases owing to the presence of strong insulin resistance and low endogenous insulin secretion in the body, which in turn leads to the development of atherosclerotic vascular lesions [34–35]. Second, hyperglycemia accelerates the progression of atherosclerosis by enhancing oxidative stress in arterial endothelial cells and the formation of advanced glycosylation end products, leading to the disruption of normal vascular endothelial function [36]. Finally, a number of cardiovascular disease risk factors, such as dyslipidemia, overweight or obesity, and hypertension, are clustered in diabetes, and a combination of these factors can lead to CVD [37–38].

### Impact on public health

Our findings have several implications for public health and research on patients with CVD. Our study points out that CVD risk already exists in prediabetes and that early identification of vascular lesions in patients living with diabetes can help reduce the excess burden of CVD complications in diabetes. Although the excess risk of coronary heart disease among patients with diabetes is largely due to diabetes-related CVD risk factors (e.g., LDL cholesterol, elevated blood pressure, and smoking) [39], the deleterious effects of the hyperglycemic state on vascular and endothelial functions are also important because of their atherosclerotic potential [40]. The association between hyperglycemia and coronary artery disease is evident in population-based studies; however, this association may not be as strong as the association between LDL cholesterol and elevated blood pressure.

### Limitations

Our study had high homogeneity among participants, a large sample size, and robustness after adjusting for various covariates. However, this study had some limitations. First, due to the limitations of the conditions, only FPG was measured during the physical examination of the residents, and glycated hemoglobin testing was not performed; therefore, we are missing this key information, which may lead to undiagnosed patients with DM or prediabetes. Second, because this study was a retrospective investigation, the introduction of unknown variables into the population or changes in known variables during follow-up may have affected the outcomes. Although we adjusted for potential confounders, we cannot rule out the existence of other known CVD risk factors (e.g., hyperlipidemia, hypertension, and other chronic diseases). Finally, because FPG was measured through population-based physical examinations, FPG values in the survey were measured only once for each participant, and fluctuations in short-term blood glucose values may have been influenced by other factors. This could have led to misclassification and a slightly inflated or reduced number of patients with diabetes in this study.

## Conclusions

In conclusion, our findings show that prediabetes and DM increase the risk of CVD compared with normal FPG levels. Abnormal fasting glucose levels in prediabetes can help identify people at high risk of CVD, emphasizing the importance early intervention in patients with diabetes to prevent CVD progression and provide an opportunity to prevent serious complications of diabetes. In future research, we need to combine glycosylated hemoglobin to reduce potential pre diabetes and patients living with diabetes.

### Electronic supplementary material

Below is the link to the electronic supplementary material.

**Supplementary Material 1: Table S1** Adjusted Hazard Ratio (95% CI) for cardiovascular disease in prediabetes, diabetes. **Table S2** Sensitivity analysis: CVD Hazard Ratio adjusted for risk factors (95% CI). **Table S3** The heterogeneity test for CVD adjusted hazard ratio with 95% CI before and after the sensitivity analysis

## Data Availability

The data sets generated and/or analysed during the current study are not publicly available due individual privacy information protection but are available from the corresponding author on reasonable request.

## Abbreviations

CI: Confidence intervals
CVD: Cardiovascular disease
DM: Diabetes mellitus
FPG: Fasting plasma glucose
IFG: Impaired fasting glucose
IGT: Impaired glucose tolerance
T2DM: Type 2 diabetes mellitus
