# Supplementary material for: The associations of risk of cardiovascular disease with development stages of diabetes in Chinese population: findings from a retrospective cohort study in QuZhou city
Source: BMC Endocr Disord. 2024 Feb 2;24:18. doi: 10.1186/s12902-024-01544-1 (PMC10835855; doi:10.1186/s12902-024-01544-1)
Supplement: Supplementary file 1 — Supplementary Material 1: Table S1 Adjusted Hazard Ratio (95% CI) for cardiovascular disease in prediabetes, diabetes. Table S2 Sensitivity analysis: CVD Hazard Ratio adjusted for risk factors (95% CI). Table S3 The heterogeneity test for CVD adjusted hazard ratio with 95% CI before and after the sensitivity analysis [file 12902_2024_1544_MOESM1_ESM.docx]

**supplementary information**

| Table S1 Adjusted Hazard Ratio (95% CI) for cardiovascular disease in prediabetes, diabetes | | | | | | | | | | |
| --- | --- | --- | --- | --- | --- | --- | --- | --- | --- | --- |
|  | N（%） | CVD（%） | Model 1 | *P* | Model 2 | *P* | Model 3 | *P* | Model 4 | *P* |
| Normal | 105310  (75.78) | 102387  (76.06) | 1.00[Ref] |  | 1.00[Ref] |  | 1.00[Ref] |  | 1.00[Ref] |  |
|  |  |  |  |  |  |  |  |  |  |  |
| Prediabetes | 18615  (13.39) | 17968  (13.35) | 1.26  (1.15,1.37) | ＜0.001 | 1.11  (1.02,1.21) | 0.015 | 1.14  (1.05,1.24) | 0.003 | 1.14  (1.05,1.24) | 0.003 |
|  |  |  |  |  |  |  |  |  |  |  |
| Diabetes | 15045  (10.83) | 14258  (10.59) | 1.90  (1.76,2.06) | ＜0.001 | 1.63  (1.50,1.76) | ＜0.001 | 1.68  (1.55,1.82) | ＜0.001 | 1.68  (1.55,1.81) | ＜0.001 |
|  |  |  |  |  |  |  |  |  |  |  |
| FPG normal was selected as the reference category. Model 1 does not adjust for covariates, Model 2 adjusts for age and sex, Model 3 adjusts for age, sex, physical activity, smoking, alcohol consumption, Model 4 adjusts for age, sex, physical activity, smoking, alcohol consumption, BMI.  *Significant results | | | | | | | | | | |

| Table S2 Sensitivity analysis: CVD Hazard Ratio adjusted for risk factors (95% CI) | | | | | | | | |
| --- | --- | --- | --- | --- | --- | --- | --- | --- |
|  | Model 1 | *P* | Model 2 | *P* | Model 3 | *P* | Model 4 | *P* |
| Exclusion of people with CVD in the first year of follow-up | | | | | | | |  |
| Normal | 1.00[Ref] |  | 1.00[Ref] |  | 1.00[Ref] |  | 1.00[Ref] |  |
| Prediabetes | 1.22(1.11,1.34)* | <0.001 | 1.09(0.99,1.19) | 0.867 | 1.11(1.01,1.22)* | 0.023 | 1.11(1.01,1.22)* | 0.024 |
| Diabetes | 1.91(1.75,2.08)* | <0.001 | 1.64(1.50,1.78)* | <0.001 | 1.69(1.55,1.84)* | <0.001 | 1.69(1.55,1.84)* | <0.001 |
| Excluding both smokers and drinkers | | | | | | | |  |
| Normal | 1.00[Ref] |  | 1.00[Ref] |  | 1.00[Ref] |  | 1.00[Ref] |  |
| Prediabetes | 1.32(1.19,1.47)* | <0.001 | 1.16(1.04,1.29)* | 0.004 | 1.19(1.07,1.32)* | <0.001 | 1.19(1.07,1.32)* | <0.001 |
| Diabetes | 2.05(1.86,2.26)* | <0.001 | 1.73(1.57,1.91)* | <0.001 | 1.79(1.62,1.97)* | <0.001 | 1.79(1.62,1.97)* | <0.001 |
| Model 1 does not adjust for covariates, Model 2 adjusts for age and sex, Model 3 adjusts for age, sex, physical activity, Model 4 adjusts for age, sex, physical activity, BMI.  *Significant results | | | | | | | | |

| Table S3 The heterogeneity test for CVD adjusted hazard ratio with 95% CI before and after the sensitivity analysis. | | | | |
| --- | --- | --- | --- | --- |
|  | Model 1 | Model 2 | Model 3 | Model 4 |
| Prediabetes for total (before sensitivity analysis) | 1.26(1.15,1.37) | 1.11(1.02,1.21) | 1.14(1.05,1.24) | 1.14(1.05,1.24) |
| Prediabetes for people without CVD in the first year of follow-up (after sensitivity analysis) | 1.22(1.11,1.34) | 1.09(0.99,1.19) | 1.11(1.01,1.22) | 1.11(1.01,1.22) |
| **Heterogeneity test: χ2 (P)** | 0.24(0.623) | 0.08(0.777) | 0.17(0.678) | 0.17(0.678) |
| Prediabetes for total (before sensitivity analysis) | 1.26(1.15,1.37) | 1.11(1.02,1.21) | 1.14(1.05,1.24) | 1.14(1.05,1.24) |
| Prediabetes for people without smoking and drinking (after sensitivity analysis) | 1.32(1.19,1.47) | 1.16(1.04,1.29) | 1.19(1.07,1.32) | 1.19(1.07,1.32) |
| **Heterogeneity test: χ2 (P)** | 0.44(0.506) | 0.39(0.530) | 0.39(0.530) | 0.39(0.530) |
| Diabetes for total (before sensitivity analysis) | 1.90(1.76,2.06) | 1.63(1.50,1.76) | 1.68(1.55,1.82) | 1.68(1.55,1.81) |
| Diabetes for people without CVD in the first year of follow-up (after sensitivity analysis) | 1.91(1.75,2.08) | 1.64(1.50,1.78) | 1.69(1.55,1.84) | 1.69(1.55,1.84) |
| **Heterogeneity test: χ2 (P)** | 0.01(0.930) | 0.01(0.918) | 0.01(0.921) | 0.01(0.920) |
| Diabetes for total (before sensitivity analysis) | 1.90(1.76,2.06) | 1.63(1.50,1.76) | 1.68(1.55,1.82) | 1.68(1.55,1.81) |
| Diabetes for people without smoking and drinking (after sensitivity analysis) | 2.05(1.86,2.26) | 1.73(1.57,1.91) | 1.79(1.62,1.97) | 1.79(1.62,1.97) |
| **Heterogeneity test: χ2 (P)** | 1.41(0.234) | 0.85(0.356) | 0.96(0.326) | 0.99(0.319) |
